# Surprisingly rich repertoire of Wnt genes in the demosponge *Halisarca dujardini*

**DOI:** 10.1186/s12862-016-0700-6

**Published:** 2016-06-10

**Authors:** Ilya Borisenko, Marcin Adamski, Alexander Ereskovsky, Maja Adamska

**Affiliations:** Department of Embryology, Faculty of Biology, Saint-Petersburg State University, Saint-Petersburg, Russia; Sars International Centre for Marine Molecular Biology, University of Bergen, Bergen, Norway; Present Address: Research School of Biology, Australian National University, Canberra, Australia; Present Address: Institut Méditerranéen de Biodiversité et d’Ecologie Marine et Continentale (IMBE), CNRS, Aix Marseille Université, IRD, Avignon Université, Marseille, France

## Abstract

**Background:**

Wnt proteins are secreted signalling molecules found in all animal phyla. In bilaterian animals, including humans, Wnt proteins play key roles in development, maintenance of homeostasis and regeneration. While Wnt gene repertoires and roles are strongly conserved between cnidarians and bilaterians, Wnt genes from basal metazoans (sponges, ctenophores, placozoans) are difficult or impossible to assign to the bilaterian + cnidarian orthologous groups. Moreover, dramatic differences in Wnt numbers among basal metazoan exist, with only three present in the genome of *Amphimedon queenslandica*, a demosponge, and 21 in the genome of *Sycon ciliatum*, a calcisponge. To gain insight into the ancestral Wnt repertoire and function, we have chosen to investigate Wnt genes in *Halisarca dujardini*, a demosponge with relatively well described development and regeneration, and a very distant phylogenetic relationship to *Amphimedon*.

**Results:**

Here we describe generation of a eukaryotic contamination-free transcriptome of *Halisarca dujardini*, and analysis of Wnt genes repertoire and expression in this species. We have identified ten Wnt genes, with only one orthologous to *Amphimedon* Wnt, and six appearing to be a result of a lineage specific expansion. Expression analysis carried out by *in situ* hybridization of adults and larvae revealed that two *Halisarca* Wnts are expressed in nested domains in the posterior half of the larvae, and six along the adult body axis, with two specific to the osculum. Strikingly, expression of one of the Wnt genes was elevated in the region undergoing regeneration.

**Conclusions:**

Our results demonstrated that the three Poriferan lineages (Demospongiae, Calcarea and Homoloscleromorpha) are characterized by highly diverse Wnt gene repertoires which do not display higher similarity to each other than they do to the non-sponge (i.e. ctenophore, cnidarian and bilaterian) repertoires. This is in striking contrast to the uniform Wnt repertoires in Cnidarians and Bilaterians, suggesting that the Wnt family composition became “fixed” only in the last common ancestor of Cnidarians and Bilaterians. In contrast, expression of Wnt genes in the apical region of sponge adults and the posterior region of sponge larvae suggests conservation of the Wnt role in axial patterning across the animal kingdom.

**Electronic supplementary material:**

The online version of this article (doi:10.1186/s12862-016-0700-6) contains supplementary material, which is available to authorized users.

## Background

Wnt genes encode secreted glycoproteins acting as signalling molecules to direct cell proliferation, migration, differentiation and survival during animal development, maintenance of homeostasis and regeneration [1–6]. While some Wnt pathway components have been identified outside of the animal kingdom, Wnt genes themselves are a conserved metazoan innovation [7, 8].

Representatives of the Wnt family have been identified in all animals studied so far, including so-called “basally branching” or non-bilaterian clades: cnidarians [9, 10], placozoans [11], ctenophores [12, 13] and sponges [14–18]. Wnt repertoires are surprisingly conserved between cnidarians and bilaterians, with 12 of 13 bilaterian orthologs present in the sea anemone, *Nematostella vectensis* [9]. This conservation appears to extend to function, as demonstrated by involvement of Wnts in segregation of germ layers during gastrulation, in embryonic and adult axial patterning and in restoration of lost body parts in both cnidarians and bilaterians [19–22]. Conservation of the blastoporal Wnt expression in cnidarians and chordates is particularly striking [5]. In cnidarian polyps such as *Hydra*, this expression persists in the oral region located in the apical part of the adult body [10]. In line with this, over-activation of Wnt signalling in *Hydra* results in formation of additional structures with head identity [23]. In chordates, where the blastopore gives rise to the anus, Wnt expression and activity confers posterior identity to developing structures [24, 25]. In line with this, over-activation of the Wnt signalling in vertebrates results in loss of anterior structures, while loss of Wnt function results in posterior truncation [26, 27].

Wnt genes identified in placozoans, ctenophores and sponges are difficult or impossible to assign to the bilaterian + cnidarian orthologous groups [12, 16–18]. Yet, Wnt expression in ctenophores and sponges is consistent with conserved involvement in axial patterning [13, 15, 18]. In particular, Wnt genes have been found to be expressed in the larval posterior pole of two major sponge model species: the demosponge *Amphimedon queenslandica* [15] and the calcisponge *Sycon ciliatum* [18]. In addition, Wnt expression is associated with osculum (the major exhalant opening of adult sponges, located at the apical pole) of *Sycon ciliatum* [18]. Such expression is consistent with homology of the larval and adult body axes between sponges and cnidarians, supporting homologous relationship between the cnidarian mouth and the sponge osculum [18, 28, 29].

While Wnt expression in adult demosponges has not been reported, pharmacological over-activation of the Wnt pathway in a freshwater species, *Ephydatia mulleri*, resulted in multiplication of the body axis, as evidenced by formation of multiple oscula [30]. This outcome is strikingly similar to Wnt over-activation experiments in cnidarians, resulting in formation of ectopic head structures [23]. Moreover, experiments involving transplantation of oscula demonstrated their organizer properties, in line with organizer properties of the cnidarian head, and animal blastopores in general [10, 31].

*Halisarca dujardini* (Chondrillida) is a marine demosponge which is very distantly related to *Amphimedon queenslandica* (Haplosclerida) [32, 33]. *Halisarca* embryonic development, metamorphosis and regeneration are well described at morphological level [34–36], but sequence resources have been lacking. Here we report generation of a transcriptome dataset and identification of a surprisingly rich Wnt repertoire (ten genes, in contrast to only three present in the genome of *Amphimedon*). Two of these genes are expressed in nested domains in the posterior half of the larvae, and six along the adult body axis, with two specific to the osculum. Moreover, Wnt expression is elevated in the region undergoing regeneration, suggesting conservation of the Wnt role in axial patterning and restoration of lost body parts across the animal kingdom.

## Methods

### Samples

No permits were required to collect sponge specimens in Norwegian waters. Total RNA and gDNA were isolated from wild-collected adult sponges and several hundred larvae freshly released in laboratory conditions. To avoid eukaryotic contaminations, the larvae were washed in sterile-filtered sea water and visually inspected under dissecting microscope. Nucleic acids were isolated using Allprep Mini kit (Qiagen) following manufacturer’s instructions, and the RNA yield and quality were determined using the NanoDrop spectrophotometer (Thermo Scientific) and the Agilent 2100 BioAnalyzer RNA 6000 Nano chip (Agilent Technologies).

### Sequencing

Two RNA-Seq libraries were prepared using Illumina TruSeq RNA Library Prep Kit: one from the wild-collected adult specimen and another one from eukaryotic contaminations-free larvae. An additional gDNA library was prepared from the same larvae using Illumina TruSeq DNA Library Prep Kit. The libraries were paired-end sequenced on Illumina HiSeq 2000 with read length of 100.

### Transcriptome assembly

The transcriptome was assembled de-novo from the two RNA-Seq libraries. The assembly was performed with Trinity 2.1.1 [37] including reads’ pre-processing with Trimmomatic [38]. We have modified Trinity’s final step called Butterfly to use read pairing information: The fasta sequence files prepared for Butterfly runs were supplemented to include both ends for all the fragments (missing-pair reads were added) and option ‘run_as_paired’ was added to all Butterfly commands. Assembled transcriptome was screened to exclude eukaryotic contaminations by aligning reads from the clean juvenile gDNA library. The alignments were done using bowtie with default parameters. Transcriptome contigs not aligned to any of the clean read were removed from the assembly. Assembly was screened for sequencing vectors using blastn against UniVec database. Transcripts of Wnt ligands were identified by sequence homology using tblastn and Wnt proteins from other organisms and are available in TSA under ids HADA01000001 – HADA01000010.

### Phylogenetic analysis

Wnt protein sequences were aligned with Mafft v7.123 using option L-INS-i. Alignment was then manually trimmed to remove poorly aligned and divergent regions. Phylogenetic tree was built using Mr Bayes 3.1 [39] which we modified to incorporate the LG model (as LG was selected as best fit substitution model by ProTest 3) [40]. Mr Bayes was run with two sets of 4 Markov chains each, till standard deviation of split frequencies dropped below 0.01.

### In situ hybridization

In situ hybridization has been carried as described for *Sycon ciliatum* [41], except that proteinase treatment was 10 min at 37 °C.

## Results and discussion

### Ten Wnt genes are present in *Halisarca dujardini*

We have generated transcriptome dataset for *Halisarca dujardini* representing genes expressed in adult specimens and free-swimming larvae (see Methods for details). Using a variety of sponge, cnidarian and bilaterian sequences we have BLAST-searched this dataset for Wnt genes and recovered ten complete coding protein sequences (Additional file 1). This stands in contrast with only three Wnt genes present in another demosponge, *Amphimedon queenslandica*, and also differs from the number of 21 genes identified in *Sycon ciliatum* (Calcarea, Calcaronea) [16, 18]. For comparison, at least eight Wnt genes are present in Homoscleromorph sponges, e.g. *Oscarella* sp. [18].

**Fig. 1:**
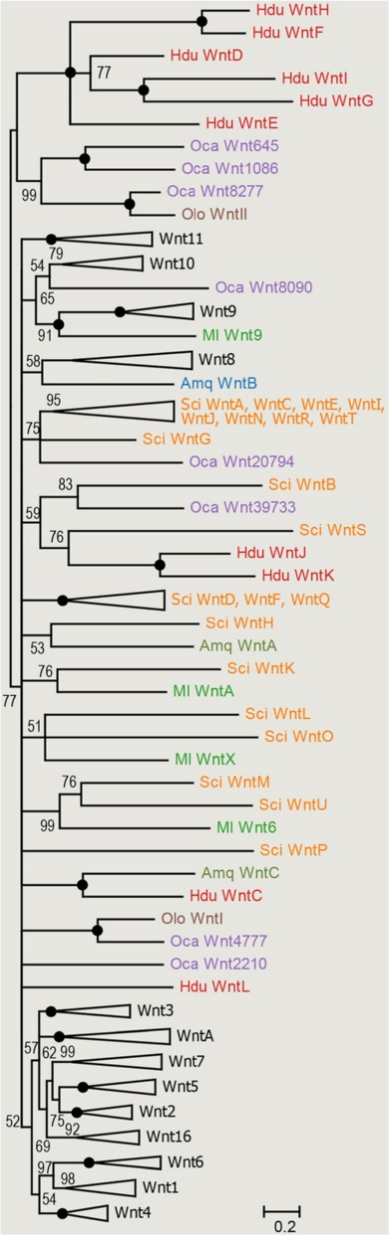
Bayesian inference gene tree of Wnt ligands. The values at the tree nodes are posterior probabilities for each split defined over the range [0, 100]. Black circles denote 100 % support (posterior probability of 1.00). Species acronyms: Amq, *Amphimedon queenslandica*, Hdu, *Halisarca dujardini*, Ml, *Mnemiopsis leidyi*, Nv, *Nematostella vectensis*, Oca, *Oscarella carmela*, Olo, *Oscarella lobularis*, Sci, *Sycon ciliatum*. Sub-trees not containing sponge sequences were collapsed; the complete tree is available as Additional file 2

We next wanted to know whether the ten newly identified *Halisarca* Wnts are orthologous to other sponge (or other metazoan) Wnt genes. We have thus carried out Bayesian analysis adding these new sequences to the previously constructed comprehensive Wnt sequence dataset [18]. Surprisingly, only one *Halisarca* sequence appeared to be in orthologous relationship with previously described Wnt genes, namely the *Amphimedon WntC* sequence, while no *AmqWntA* or *AmqWntB* orthologues could be identified in *Halisarca* (Fig. 1 and Additional file 2). We have named this gene *HduWntC*, and the remaining nine sequences *HduWntD-WntL* according to the order in which they were identified. Of these, six clustered together in our analysis with high support, suggesting they are likely a result of independent subfamily expansion in the *Halisarca* lineage (Fig. 1). Another pair of *Halisarca* Wnts was associated with *Sycon ciliatum WntS*, although with very weak support, and the last one showed no particular affinity to any of previously identified Wnt genes (Fig. 1).

Thus, all so-far studied sponges, representing three Poriferan lineages (Demospongiae, Calcarea and Homoloscleromorpha) are characterized by highly diverse Wnt gene repertoires which do not display higher similarity to each other than they do to the non-sponge (i.e. ctenophore, cnidarian and bilaterian) repertoires. This is in striking contrast to the uniform Wnt repertoires in Cnidarians and Bilaterians, suggesting that the Wnt family composition became “fixed” only in the last common ancestor of Cnidarians and Bilaterians.

### *Halisarca* Wnts are expressed along the adult and larval axes and during regeneration

In ctenophores, cnidarians and calcareous sponges Wnt genes are expressed along the major (oral-aboral or apical-basal) body axis in sets of nestled domains, suggesting existence of a “Wnt code” possibly conveying positional information [4, 9, 10, 13, 18]. In *Oscarella lobularis*, a homoscleromorph sponge, two Wnt genes are expressed in a complementary fashion with domains in the ostia (multiple openings in the inhalant canals on the surface of the body) and in exopinacoderm surrounding the ostia of adult specimens [17]. It is important to note here that at least six other Wnt genes are present in *Oscarella* sp. [18], expression of which has not been reported so far.

**Fig. 2:**
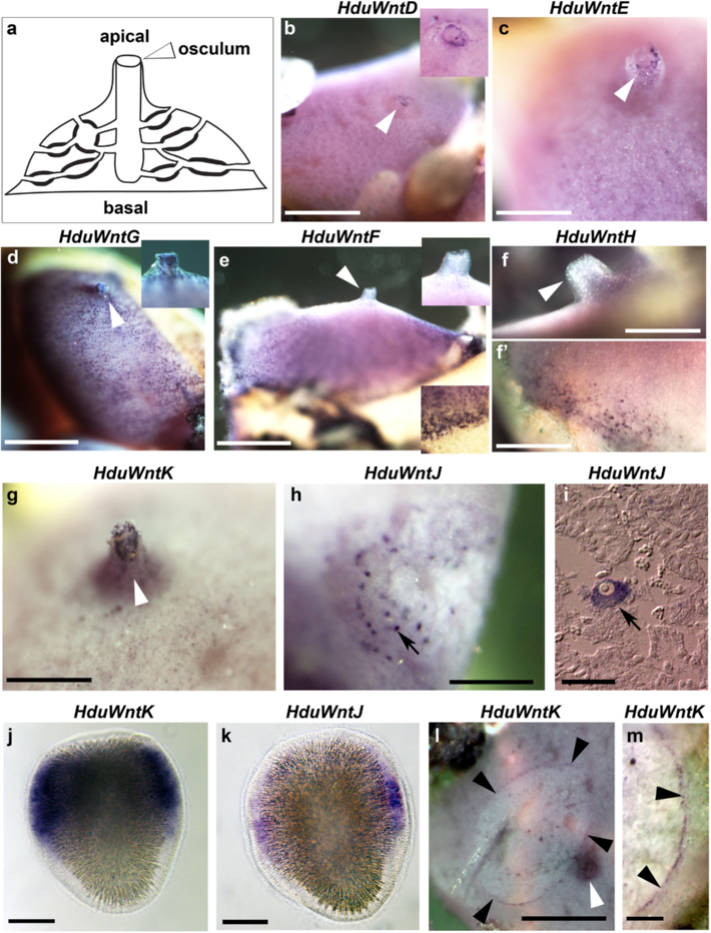
Expression of Wnt genes in *Halisarca dujardini*. **a** schematic representation of the adult body plan of *Halisarca*, apical and basal regions and the osculum are labelled; **b**, **c**, *HduWntD* and *HduWntE* transcripts are localized around the osculum; **d**, *HduWntG* transcripts are present throughout the exopinacoderm and particularly in the oscular chimney; **e**, **f**, **f’**, *HduWntF* and *HduWntH* transcripts are absent from the osculum and the apical region, but strong along the base; **g**, *HduWntK* transcripts are present along the oscular chimney; **h** and **i**, *HduWntJ* transcripts are present in the oocytes; **j**, *HduWntK* transcripts are present in the posterior half of the larva except the polar region; **l**, *HduWntJ* transcripts are present in cells distributed along the larval equator; **l** and **m**, *HduWntK* transcripts are conspicuously present along the wound margin. White arrowheads indicate the osculum; insets in the upper and lower corners are enlargements of the apical and basal regions, respectively, black arrowheads indicate wound margin; black arrows indicate oocytes; posterior pole of the larvae is towards the top. Note that the specific staining is dark purple, while the uniform pink coloration of some samples is background staining. Scale bars: **b**, **d**, **e** – 5 mm; **c** – 2.5 mm; **f**, **f’** – 2 mm; **g**, **h**, **l**, **m** – 3 mm; **i** – 30 μm; **j**, **k** – 50 μm

We have attempted cloning and expression analysis of all ten *Halisarca* Wnts. Of these, six genes were expressed in the adult specimens in four unique patterns:

*WntD* (Fig. 2b) and *WntE* (Fig. 2c) at the tip of the osculum; *WntG* (Fig. 2d) throughout the entire exopinacoderm, with particularly high concentration of positive cells in the osculum; *WntF* (Fig. 2e) and *WntH* (Fig. 2f, f’) in the peripheral exopinacoderm and basopinacoderm; and finally *WntK* (Fig. 2g, see also Fig. 2l) was prominently expressed within the oscular chimney, and weakly throughout the exopinacoderm.

Detection of *WntJ* expression revealed positive large cells within the mesohyl of some specimens, which were identified as young oocytes upon sectioning (Fig. 2h, i). While during our collections we have not found any specimens with embryos, we have been able to carry out *in situ* hybridization on larvae released from adults briefly maintained in laboratory conditions. Two *Halisarca* Wnt genes revealed robust expression in the larvae: *WntK* (Fig. 2j) throughout most of the posterior hemisphere, except of the polar cells themselves, and *WntJ* in a band of equatorial cells (Fig. 2k).

Thus, the identified Wnt expression domains encompass the entire apical-basal axis of the adult *Halisarca* body, with majority of the genes expressed uniquely or predominantly in the osculum. At the same time, Wnt expression is associated with the posterior region of the larvae. These nested patterns, and the prevalence of apical and posterior expression are consistent with the postulated conservation of Wnt role in axial patterning throughout the metazoans.

In addition to the conserved role in axial patterning, Wnt genes are also known to be involved in wound healing and regeneration in many animal lineages [3, 6, 10, 19–22]. We have recently described cellular processes leading to regeneration of the ectosome in *H. dujardini* [36], and we wanted to know whether Wnt genes might be involved in these processes. While majority of the Wnt genes did not display detectable expression changes in the regeneration zone, *HduWntK* expression was prominent in the exopinacocytes surrounding the wound at 12 h after wounding (Fig. 2l, m). These cells are actively involved in the regeneration, as they temporarily dedifferentiate, phagocyte the debris and contribute to restoration of the ectosome by migration and re-differentiation [36]. Thus, as in other animal lineages, the demosponge Wnt pathway is implicated in the regeneration processes.

## Conclusions

Transcriptome sequencing of *Halisarca dujardini* allowed us the first insight into gene repertoire of a demosponge from a previously unexplored order. We have identified ten Wnt genes, nine of which are without orthologs in any previously reported species. While the diversity of Wnt subfamilies is striking, expression of the identified genes suggests conservation of roles in axial patterning and regeneration. We wonder what mechanisms are responsible – or permissive – for the apparent lack of constraints on Wnt protein sequences in sponges (as well as ctenophores and possibly placozoans) as opposed to cnidarians and bilaterians.

## Abbreviations

*Amq*, *Amphimedon queenslandica*; BLAST, Basic Local Alignment Search Tool; ENA, European Nucleotide Archive; gDNA, genomic deoxyribonucleic acid; *Hdu*, *Halisarca dujardini*; LG model, Le-Gascuel substitution model; *Ml*, *Mnemiopsis leidyi*; *Nv*, *Nematostella vectensis*; *Oca*, *Oscarella carmela*; *Olo*, *Oscarella lobularis*; RNA, ribonucleic acid; *Sci*, *Sycon ciliatum*; SRA, Sequence Read Archive; TSA, Transcriptome Shotgun Assembly.

## Additional files

Additional file 1: wnt.alignment.nex.txt: trimmed alignment of Wnt protein sequences used to generate Bayesian inference trees shown in Additional file 1 and Fig. 1. Species: Amq, *Amphimedon queenslandica*, Ate, *Achaearanea (Parasteatoda) tepidariorum*, Bf, *Branchiostoma floridae*, Cte, *Capitella teleta*, Hdu, *Halisarca dujardini*, Hs, *Homo sapiens*, Lgi, *Lottia gigantea*, Ml, *Mnemiopsis leidyi*, Nv, *Nematostella vectensis*, Oca, *Oscarella carmela*, Olo, *Oscarella lobularis*, Sci, *Sycon ciliatum*, Sko, *Saccoglossus kowalewski*, Spu, *Strongylocentrotus purpuratus*, Tc, *Tribolium castaneum*. (TXT 39 kb) Additional file 2: wnt.tree.nwk.txt: Newick format Bayesian inference gene tree of Wnt ligands. Species: Amq, *Amphimedon queenslandica*, Ate, *Achaearanea (Parasteatoda) tepidariorum*, Bf, *Branchiostoma floridae*, Cte, *Capitella teleta*, Hdu, *Halisarca dujardini*, Hs, *Homo sapiens*, Lgi, *Lottia gigantea*, Ml, *Mnemiopsis leidyi*, Nv, *Nematostella vectensis*, Oca, *Oscarella carmela*, Olo, *Oscarella lobularis*, Sci, *Sycon ciliatum*, Sko, *Saccoglossus kowalewski*, Spu, *Strongylocentrotus purpuratus*, Tc, *Tribolium castaneum*. (TXT 3 kb)
